# Caloric Restriction and Dietary Taurine Regulate Taurine Homeostasis Through Distinct Tissue‐Specific Mechanisms in Mice

**DOI:** 10.1002/mnfr.70414

**Published:** 2026-02-12

**Authors:** András Gregor, Arturo Auñon‐Lopez, Qendrim Zebeli, Marc Pignitter, Kalina Duszka

**Affiliations:** ^1^ Department of Nutritional Sciences University of Vienna Vienna Austria; ^2^ Institute of Physiological Chemistry Faculty of Chemistry University of Vienna Vienna Austria; ^3^ Vienna Doctoral School in Chemistry (DoSChem) Faculty of Chemistry University of Vienna Vienna Austria; ^4^ Centre For Animal Nutrition and Welfare University of Veterinary Medicine Vienna Austria

**Keywords:** caloric restriction, glutathione, intestine, liver, taurine

## Abstract

Caloric restriction (CR) stimulates taurine‐conjugated bile acids (BA) synthesis in the liver. Upon secretion into the intestine, BAs undergo deconjugation, increasing taurine, and taurine conjugate levels, including taurine‐glutathione (GSH). This study aimed to determine whether dietary taurine and CR‐induced taurine changes operate through distinct regulatory mechanisms. Male C57Bl/6 mice were subjected to ad libitum feeding or 20% CR with low‐taurine diet (LTD) or 5% taurine in drinking water. LTD and taurine supplementation minimally affected intestinal taurine concentrations and did not disrupt CR‐induced changes in intestinal taurine levels, GSH conjugates, and GST expression, demonstrating mechanistic independence. Both interventions significantly altered hepatic and plasma taurine levels, indicating tissue‐specific regulation. While CR primarily influenced GSH‐S transferase (GST) mRNA expression in the intestine, GST activity correlated with substrate availability rather than gene expression. CR maintained enhanced intestinal taurine retention during taurine supplementation, evidenced by reduced fecal taurine excretion compared to controls. Dietary and CR‐related taurine are affected by distinct tissue‐specific mechanisms, with CR primarily impacting intestinal taurine while modulation of dietary taurine (restriction or supplementation) predominantly influences hepatic pools. The study reveals independent regulatory mechanisms governing taurine homeostasis and emphasizes differences between dietary factors and physiological responses during CR.

## Graphical abstract

Caloric restriction and dietary taurine each regulate taurine homeostasis independently. The intestinal taurine pool is predominantly affected by synthesis triggered by caloric restriction, whereas hepatic taurine levels vary according to dietary intake.

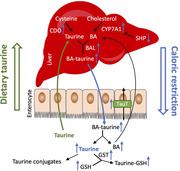

**Abbreviations**
BA: Bile acids
CR: Caloric restriction
GI: Gastrointestinal
GSH: Glutathione
GST: GSH‐S Transferases
LTD: Low‐taurine diet
SPF: Standard specific‐pathogen‐free

## 1 Introduction

Taurine is a free amino acid that mammals obtain from both dietary sources and biosynthesis. It is present in a wide array of natural food sources, particularly high in meat, fish, and seafood, dairy products, eggs, and algae [1]. Notably, relatively high amounts of taurine are added to various energy drinks and supplements [2]. Upon ingestion, taurine is transported across intestinal epithelial cells, then carried through the bloodstream and absorbed into cells in target tissues with the participation of the taurine transporter Slc6a6. Whereas taurine biosynthesis primarily occurs in the liver, brain, and kidneys, with synthesis and turnover rates varying among different animal species [3, 4, 5]. Sulfur amino acids such as methionine and cysteine serve as substrates for taurine biosynthesis, and their availability regulates catalytic efficiency and degradation of cysteine dioxygenase (CDO), one of the enzymes required for taurine biosynthesis [1, 3, 4, 5].

Taurine is highly abundant and particularly concentrated in various organs and tissues, including the intestine, kidneys, liver, and bile, as a conjugate of bile acids (BA). BA are a group of cholesterol derivatives with amphiphilic properties and diverse structures. The biosynthesis of BAs occurs in the liver and the classical pathway is initiated by the rate‐limiting enzyme cholesterol 7α‐hydroxylase (CYP7A1) [6, 7]. Nuclear receptor small heterodimer partner (SHP) represses Cyp7a1 expression, thereby inhibiting BA synthesis. Following their generation, BAs undergo conjugation with taurine or glycine [6, 8]. The ratio of conjugation with either of the amino acids may be affected by the diet. However, on average, in mice, 95% of BAs are conjugated to taurine and 5% to glycine, and in humans, 70% to glycine and 30% to taurine [7]. The conjugation of taurine is catalyzed by enzymes BA CoA‐ligase (BAL) and BA CoA:amino acid N‐acyltransferase (BAT) [6, 7].

Upon meal ingestion, BAs are secreted in the intestine to aid solubilization, digestion, and absorption of lipid and lipid‐soluble vitamins [6, 8]. However, we reported that caloric restriction (CR) increases hepatic BA synthesis, conjugation, and release into the gastrointestinal (GI) tract of mice [9, 10, 11]. Following secretion into the intestine, BAs undergo modification by microbial bile salt hydrolase (BSH), leading to the release of deconjugated BAs and free taurine [12]. Next, the majority of BAs are further metabolized to secondary BAs, taken up, and recirculated to the liver. Notably, the intestines of CR animals show enhanced capacity to absorb BAs, leading to reduced loss of BAs with feces [10].

As we showed [9], the taurine released upon BA deconjugation creates conjugates with various molecules. Notably, the majority of the taurine conjugates, and thus, their potential role, remain uncharacterized [9, 12, 13]. So far, we have identified chlorotaurine and taurine‐glutathione (GSH) [9]. The occurrence of taurine‐GSH is accompanied by an elevated expression and activity of GSH S‐transferase (GST) [9, 12, 13, 14]. GSTs are a family of enzymes that catalyze the conjugation of GSH to different electrophilic substrates. The generated water‐soluble conjugates are further directed to excretion via urine and bile [15]. So far, we have found that taurine‐GSH conjugate supports taurine uptake from the intestine in CR animals. This results in lower levels of taurine secreted in the feces of CR mice compared to ad libitum‐fed controls [9, 13]. The absorbed taurine is then distributed within the body, and CR impacts its uptake to several organs, including the kidney, heart, spleen, and adipose tissue [13, 16]. There, taurine plays various roles, for example, contributes to CR‐triggered adipose tissue loss by stimulating β‐oxidation [16, 17, 18].

Taurine homeostasis during CR represents a poorly understood mechanism that may contribute to CR's beneficial effects on healthspan and lifespan extension [19]. Our previous studies have established that CR increases taurine levels in intestinal mucosa through enhanced BA synthesis, deconjugation, and subsequent taurine‐glutathione conjugate formation [12]. However, the specific contribution of dietary taurine to this CR‐induced phenotype remains unclear.

Given that dietary taurine supplementation and CR share overlapping benefits, including improved mitochondrial function [19, 20, 21, 22], reduced oxidative stress [20, 22], anti‐inflammatory impact [11, 23, 24, 25], improved cardiovascular, [26, 27, 28] and neurological health [29, 30], support for body weight loss and diabetes treatment [16, 31, 32, 33], as well as life‐extending properties [19, 34, 35], we hypothesized that dietary taurine modulation could either enhance or disrupt the CR‐induced taurine homeostasis changes. Specifically, we hypothesized that: (1) dietary taurine supplementation would enhance CR‐induced changes in taurine concentrations and related metabolic markers, and (2) restriction of dietary taurine and its precursors (cysteine and methionine) would attenuate the CR‐induced elevation of taurine levels and associated enzymatic activities.

To test these hypotheses, we challenged the CR‐induced elevation of intestinal taurine and its conjugates bidirectionally using both a low‐taurine diet (LTD) and taurine supplementation, measuring established CR‐related parameters including taurine concentrations, taurine‐glutathione conjugates, GST enzyme activity, and BA profiles across liver, intestinal mucosa, plasma, and fecal compartments.

## 2 Experimental Section

### 2.1 Animal Care and Experimental Procedures

Male C57Bl/6 mice were purchased from Janvier Labs (Le Genest‐Saint‐Isle, France) and housed under a 12‐h light/12‐h dark cycle in standard specific‐pathogen‐free (SPF) conditions. Mice aged 10 weeks were randomly assigned to experimental groups as control ad libitum‐fed or CR mice. Ad libitum control animals were kept in standard conditions in group cages (4 mice per cage). While the mice in the CR group were fed once a day with a specified amount of chow, corresponding ∼80% of their ad libitum food intake. This restriction lasted for 14 days, during which the mice were housed in individual cages to prevent aggression and ensure equal distribution of food portions. This protocol corresponds to the restriction applied in our previous studies, in which an increase in taurine levels was discovered and confirmed [9, 12, 13, 14, 16, 36].

To restrict dietary taurine intake, ad libitum and CR groups were given a LTD or an isocaloric matched control diet (table S1) as previously published [16]. The diets were custom‐made by SSNIFF‐Spezialdiäten GmbH (Soest, Germany). The amount of LTD given to CR animals was calculated based on the volountary in the in the ad libitum group.

In the second experiment, the animals were fed a V153x R/M‐H auto diet (table S2) from SSNIFF‐Spezialdiäten GmbH (Soest, Germany). One ad libitum and one CR group served as controls, and another ad libitum and CR group received 5% taurine (≥99%, Cat no. T8691, Merck KGaA, Darmstadt, Germany) in drinking water.

The diets were not micronutrient‐matched between CR and ad libitum groups. This design reflects established practice in short‐term CR studies and takes into account that basal chow exceeds minimal micronutrient requirements [80] and that 20% restriction over 14 days is unlikely to cause deficiencies [81]. Moreover, this protocol mirrors natural CR conditions without supplementation and, for continuity, matches the protocols used in our previous studies.

The food was removed from cages 2 h before the dissection. Mice were euthanized via isoflurane (Dechra Pharmaceuticals plc, Northwich, UK) overdose and a cardiac puncture. Liver, ileum mucosa, plasma, and feces were snap‐frozen in nitrogen and stored at −80°C. All the organs were collected between 9 am and 11 am.

The ARRIVE guidelines were followed during the design and performance of the experiments. All animal experimentation protocols were approved by the Bundesministerium für Wissenschaft, Forschung und Wirtschaft, Referat für Tierversuche und Gentechnik (BMWFW‐66.006/2020 and 2022‐0.257.032). All experiments were performed in accordance with the Animal Welfare Act guidelines for animal experimentation.

#### 2.1.1 Dosage

In experiment 2, taurine was administered in drinking water as 5% solution for the entire duration of the experiment. The animals consumed approximately 4.5 mL of water per day, corresponding to ca. 220 mg of taurine.

### 2.2 GSH, Taurine, and Taurine Conjugates Detection

Samples were processed following a protocol previously published [9, 12, 14]. Briefly, 7–10 mg of the sample was placed in Precellys tubes containing 1.4 mm ceramic beads, and nine times the volume of absolute methanol at −20°C was added. The samples underwent homogenization using the Precellys24 Tissue Homogenizer (Bertin Instruments, Montigny‐le‐Bretonneux, France) for two rounds of 15 s each at 5000 rpm, followed by vortexing for 30 s and incubation at −20°C. Subsequently, the samples were centrifuged for 10 min at 18000 × *g*, and the supernatants were transferred to new tubes. This centrifugation step was repeated, and the supernatants were then transferred into HPLC vials using a thermostatic autosampler maintained at 4°C.

The samples underwent LCMS analysis in negative mode with an LCMS‐8040 Liquid Chromatograph Mass Spectrometer (Shimadzu Corporation, Kyoto, Japan) equipped with an Atlantis T3 3 µm column (2.1 x 150 mm, Waters, Milford, MA, USA). The column temperature was held at 40°C. Eluent A contained 0.1% formic acid in water, whereas eluent B consisted of 0.1% formic acid in acetonitrile. The gradient began at 5% B for 2.5 min, increased to 20% B by 8 min, and returned to 5% B at 9 min with a 1‐min hold. The area under the curve (AUC) for each molecule was quantified and utilized for sample comparison.

### 2.3 Bile Acid (BA) Detection

For BA detection, previously reported methods were followed [9, 10, 37]. Briefly, intestinal mucosa and liver samples were homogenized in Precellys tubes with ceramic beads using nine volumes of ice‐cold 100% methanol. After homogenization, the samples were shaken on ice for 10 min, vortexed, and centrifuged for 10 min at 12000 g and 4°C. The supernatants were then transferred to HPLC vials and stored at 4°C until analysis. Analysis was performed in positive mode using an LCMS‐8040 Liquid Chromatograph Mass Spectrometer (Shimadzu Corporation, Kyoto, Japan) with an Atlantis T3 3 µm column (2.1x150 mm, Waters, Milford, MA, USA). Solvent A consisted of water with 0.1% formic acid and 20 mmol/L ammonium acetate, while solvent B included acetonitrile/methanol (3/1, v/v) with 0.1% formic acid and 20 mmol/L ammonium acetate. The solvent gradient began at 30% B for 5 min, reached 100% B by 25 min, and maintained this level for 20 min. For re‐equilibration, the mix was reverted back to the initial 30% B ratio for 10 min.

### 2.4 GST Activity Assay

GST activity was assessed using commercial assay kits as per the manufacturer's guidelines (Cat no. CS0410, Merck KGaA). This assay measures total GST activity using 1‐chloro‐2,4‐dinitrobenzene (CDNB) as substrate, detecting GST isoforms including GSTA, GSTM, and GSTP classes. Tissue homogenates (10‐20 mg) were prepared in phosphate buffer (pH 6.5), and activity was measured spectrophotometrically at 340 nm. Results are expressed as µmol/min/mg protein, normalized to total protein content

### 2.5 Gene Expression Analysis

For gene expression analysis, RNA was extracted from intestinal mucosa with the RNeasy mini kit (Qiagen, Hilden, Germany). Samples were thawed in lysis buffer, disrupted using a syringe and needle, and processed according to the manufacturer's instructions. Reverse transcription was conducted with SuperScript II Reverse Transcriptase (InvitrogenTM, Life Technologies, Carlsbad, CA, USA). Quantitative real‐time PCR (qRT‐PCR) was performed using the QuantStudioTM 6 Flex Real‐Time PCR System and SYBR Green PCR Master Mix (both from Applied Biosystems, Life Technologies, Carlsbad, CA, USA). The results shown represent the mean ΔΔCt for each experimental group, with *Eef1a1* as the reference. The primer sequences are listed in the Table S3.

### 2.6 Statistics

Two‐way ANOVA with Bonferroni correction was employed to assess statistical significance.

Heatmaps were created to show the levels of taurine conjugates across different experimental groups, utilizing Z‐Scored data that reflects deviations from the group mean values, arranged through hierarchical clustering. These heatmaps were visualized with the COVAIN extension (v2019.04) in MATLAB (R2018b).

## 3 Results

**FIGURE 1:**
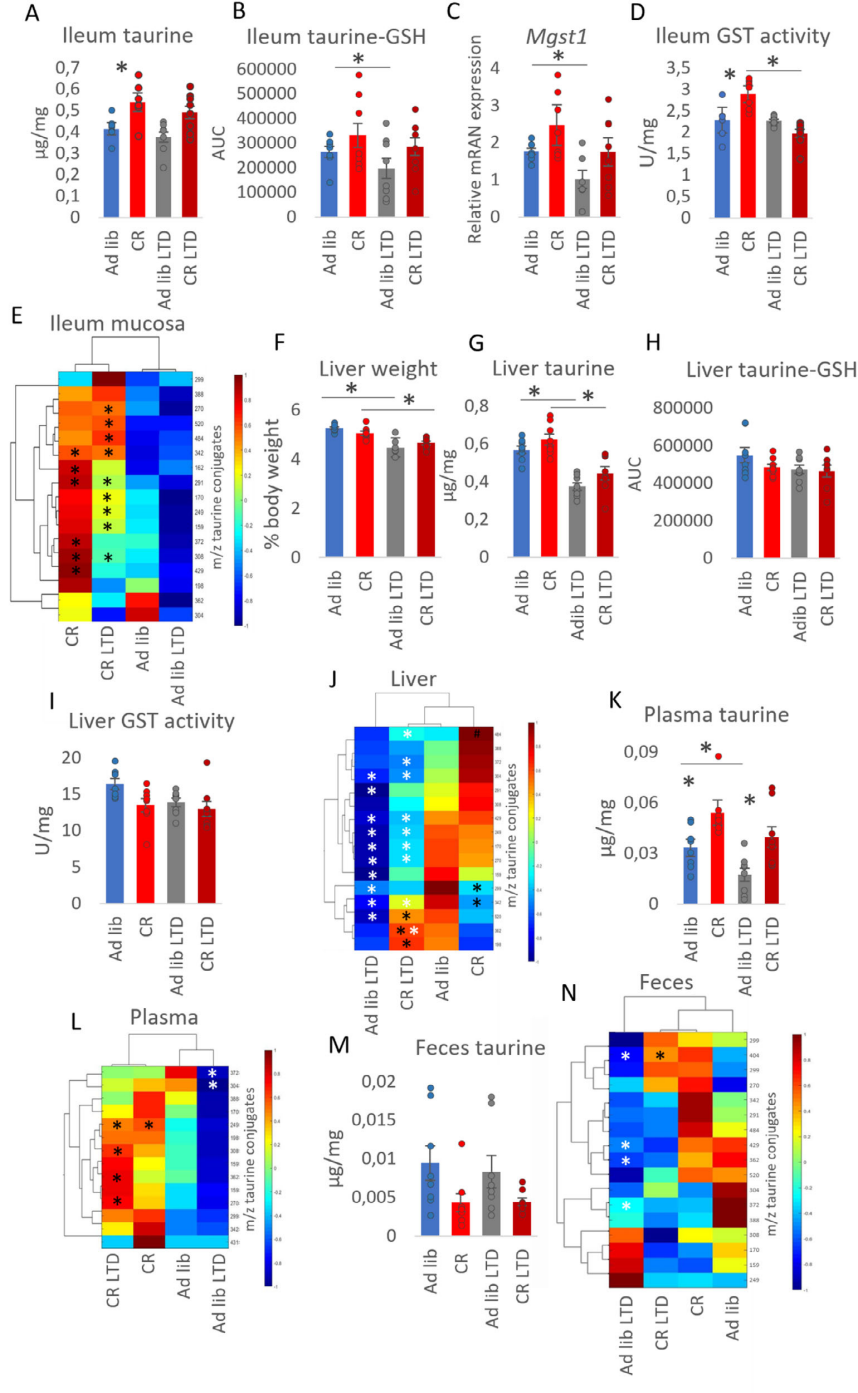
Restriction of dietary taurine impacts mainly taurine levels in the liver. Male mice were subjected to ad libitum feeding (Ad lib) or caloric restriction (CR) and given a control or low‐taurine diet (LTD). Free taurine (A) and taurine‐glutathione (GSH) conjugate levels (B) as well as mRNA expression of *Mgst1* (C) and the enzymatic activity of GSH‐S transferases (D) were determined in ileum mucosa. The heatmap displays the levels of various taurine conjugates in the ileum mucosa (E). The weight of the liver was recorded and displayed as a percentage of body weight (F). Free taurine concentrations (G), taurine‐GSH conjugate levels (H), the enzymatic activity of GSH‐S transferases (I), and the levels of multiple taurine conjugates (J) were measured in the liver. Free taurine (K) and taurine conjugates (L) levels were measured in plasma. Similarly, the concentration of free taurine (M) and the levels of taurine conjugates were determined in feces (N). In the heatmaps, the *y*‐axis represents the m/z ratio of each taurine conjugate species, while the *x*‐axis indicates the experimental groups. Color intensity represents Z‐scored abundance values, as shown in the scale bar. Group ordering in heatmaps is determined by hierarchical clustering analysis based on the similarity of metabolite profiles. ANOVA with Bonferroni correction was applied to assess statistical differences between the groups. * indicates statistical significance. In the heatmaps, statistical significance marked in black indicates the difference between control (Ad lib lib vs. CR) or LTD (Ad lib LTD lib vs. CR LTD) groups, while statistical significance marked in white indicates the difference between Ad lib (Ad lib vs. Ad lib) LTD or CR (CR vs. CR LTD) groups. Bars represent the mean of seven to eight biological replicates ±SEM.

To challenge the CR‐related increase in taurine levels in the intestinal mucosa of CR mice, both ad libitum‐fed and CR animals were subjected to a diet low in taurine and its precursors cysteine and methionine (referred to as LTD). The body weight loss was higher in the CR than the CR LTD group but did not differ between the Ad lib and ad lib LTD mice (figure S1A). As in our previous studies [9, 13], CR increased taurine concentration in the mucosa of the ileum (Figure 1A). The LTD diet had negligible or minimal effects on taurine concentration in the intestinal mucosa (Figure 1A). However, the levels of taurine‐GSH conjugate were reduced in Ad lib LTD group compared to Ad lib animals (Figure 1B). Correspondingly, the mRNA expression of *Mgst1*, one of the GSTs, decreased in the Ad lib LTD group (Figure 1C), and the enzymatic activity of GST was reduced in CR LTD compared to CR (Figure 1D).

Besides taurine‐GSH, the levels of other taurine conjugates were measured in the ileum mucosa (Figure 1E). Both CR and CR LTD groups showed an increase in the levels of several conjugates and a comparison between CR LTD and Ad lib LTD revealed that LTD did not neutralize the CR‐triggered increase in the levels of taurine conjugates (Figure 1E). The LTD groups tended to differ from the corresponding chow‐fed groups; however, there were no statistically significant differences (Figure 1E).

Next, the impact of LTD was assessed in the liver. The weight of the liver was affected by CR as well as LTD, with CR and Ad lib LTD mice liver weighing less than those of Ad lib animals (figure S1B). When accounting for body weight differences, the liver in Ad lib LTD and CR LTD animals was lighter than in the corresponding control Ad lib and CR groups (Figure 1F). Consistent with our previous findings [9, 13], CR did not affect hepatic taurine or taurine‐GSH levels (Figure 1G,H). However, taurine concentration was reduced in both Ad lib LTD and CR LTD compared to their corresponding chow‐fed controls (Figure 1G). Whereas the levels of taurine‐GSH or the activity of GST transferases in the liver were not affected by either CR or LTD (Figure 1H‐I).

Corresponding to the changes in taurine concentration, the levels of multiple taurine conjugates were decreased in the liver of LTD mice compared to corresponding controls (statistical significance marked in white) (Figure 1J).

As in our previous experiments [10], CR increased levels of several types of BAs in the liver (figure 1C). Overall, LTD diet did not substantially affect BA levels and composition in the liver. However, it neutralized the impact of CR for taurolithocholic acid (TLCA), cholic acid (CA), and taurodeoxycholic acid (TDCA) (figure 1C).

In the plasma, taurine concentration was reduced in the Ad lib LTD compared to Ad lib (Figure 1K). Both CR groups exhibited increased concentrations of taurine compared to their corresponding Ad lib groups (Figure 1K). Regarding taurine conjugates, the levels of two of them were reduced in Ad lib LTD compared to Ad lib (statistical significance marked in white) and increased in CR LTD compared to Ad lib LTD (statistical significance indicated in black) (Figure 1L). Comparing CR and CR LTD animals, no statistically significant differences were observed in the levels of taurine conjugates (Figure 1L).

In a previous report [9], we showed that CR reduced taurine concentration in the feces. In the case of the current data set, the differences were not statistically significant due to high variability in the CR groups (Figure 1M). Concerning taurine conjugates, four of them were statistically significantly downregulated in Ad lib LTD compared to LTD (statistical significance indicated in white), and one was reduced in Ad lib LTD compared to CR LTD (statistical significance marked in black) (Figure 1N).

**FIGURE 2:**
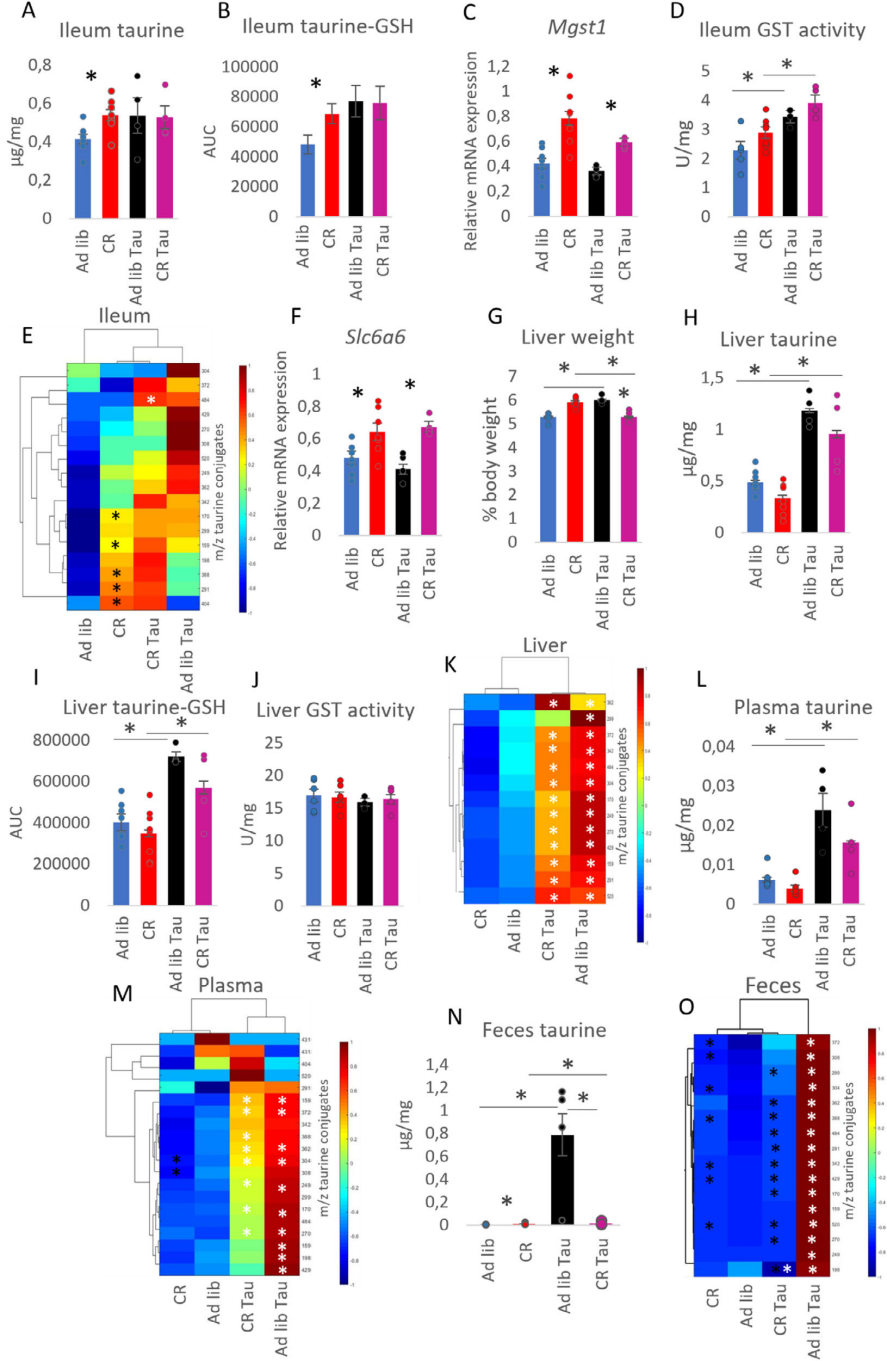
Supplementation of taurine primarily affects taurine levels in the liver, plasma, and feces, with CR introducing a difference in the fecal taurine levels. Male mice were subjected Ad lib or CR. Half of the animals from each group were given 5% taurine in drinking water (Tau). Free taurine concentration (A), taurine‐GSH conjugate levels (B), the mRNA expression of *Mgst1* (C), the enzymatic activity of GSH‐S transferases (D), taurine conjugate levels (E), and taurine transporter *Slc6a6* gene expression (F) were assessed in the ileum mucosa. The weight of the liver was measured and shown as a percentage of body weight (G). In the liver, free taurine concentrations (H), taurine‐GSH conjugate levels (I), the enzymatic activity of GSH‐S transferases (J), and the levels of various taurine conjugates were measured (K). Free taurine concentrations (L) and the levels of taurine conjugates (M) were measured in plasma. Similarly, free taurine (N) and taurine conjugate levels (O) were determined in feces. In the heatmaps, the y‐axis displays the m/z ratio for each taurine conjugate species, whereas the x‐axis indicates the experimental groups. Color intensity indicates Z‐scored abundance values and group ordering in heatmaps is based on hierarchical clustering analysis of metabolite profiles similarity. Heatmap groupings are organized through hierarchical clustering according to metabolite profile similarities. ANOVA was applied to assess statistical differences between the groups. * represents statistical significance. In the heatmaps, statistical significance marked in black indicates the difference between the Ad lib and CR groups, or between the Ad lib LTD and CR LTD groups. Meanwhile, statistical significance marked in white indicates the difference between the Ad lib and Ad lib LTD groups, or between the CR and CR LTD groups. Ad lib and CR *n*=8, Ad lib Tau and CR Tau *n*=4. Error bars represent ±SEM.

To further challenge the CR‐induced increase in intestinal taurine and taurine conjugates, Ad lib, and CR mice were given 5% taurine in drinking water. The CR animals from both groups lost similar amounts of body weight (figure 1D). Taurine supplementation neutralized the difference in ileum mucosa taurine concentration typically found between the Ad lib and CR groups. Namely, the intervention increased taurine levels in the Ad lib Tau group to levels comparable to the CR group; however, it did not result in any statistically significant changes. (Figure 2A).

Changes in taurine‐GSH conjugate levels in the ileum mucosa mirrored the pattern seen for taurine concentration in the same tissue (Figure 2B), much like the case of LTD (Figure 1B). Taurine‐GSH levels were higher in CR compared to the Ad lib group, and taurine supplementation disturbed this pattern. Taurine intake raised taurine‐GSH levels in the Ad lib group but did not further increase it in CR Tau group (Figure 2B). Notably, the mRNA expression pattern of *Mgst1* did not overlap with taurine‐GSH levels but was modulated by CR (Figure 2C). To clarify this discrepancy, GSTs activity was assessed. Supplementation with taurine increased GST activity in both Ad lib Tau and CR Tau groups (Figure 2D), and it corresponded to taurine‐GSH levels more closely than GSTs mRNA expression pattern (Figure 2C), implying that GST activity and taurine‐GSH levels were regulated by taurine levels, but the gene expression was stronger influenced by CR.

CR led to increased levels of a few taurine conjugates in the ileum mucosa compared to Ad lib (statistical significance marked in black) (Figure 2E). Supplementation of taurine tended to increase levels of taurine conjugates; however, only one conjugate was statistically significantly affected in CR Tau compared to CR (Figure 2E).

The expression of taurine transporter *Slc6a6* maintained the Ad lib‐CR pattern in the ileum of Tau animals, indicating a stronger impact of CR compared to taurine supplementation on gene expression (Figure 2F).

Next, taurine supplementation increased liver weight in Ad lib Tau compared to Ad lib but decreased it in CR Tau compared to CR when considering absolute organ weight (Figure S1E) and relatively to total body weight (Figure 2G).

Hepatic concentrations of taurine were stronger affected by taurine supplementation than intestinal concentrations in both Ad lib Tau and CR Tau groups (Figure 2H). The levels of taurine‐GSH closely followed changes in taurine concentration (Figure 2I), even though the activity of GSTs was unaffected (Figure 2I), similar to what was observed in the liver of LTD animals (Figure 1I).

A marked increase in the levels of taurine conjugates was measured in Ad lib Tau and CR Tau compared to their control Ad lib and CR groups (statistical significance marked in white) (Figure 2J). Contrary, no differences in taurine conjugates were observed between the liver of Ad lib and CR groups for both control and Tau conditions (Figure 2J), corresponding to our previous report [9].

Regarding hepatic BA, TCA concentration increased in the Ad lib Tau versus the Ad lib group (Figure S1F). Additionally, a similar trend was measured for tauroursodeoxycholic acid (TUDCA). Correspondingly to the slight increase in certain BAs levels, the mRNA expression of the *Cyp7a1* gene was higher in Ad lib Tau compared to the Ad lib group (Figure S1G). Whereas taurine supplementation did not affect *Shp* gene expression (Figure S1G). Finally, genes involved in taurine biosynthesis and conjugation, *Cdo* and *Bal*, were modestly affected by dietary taurine (Figure S1G). In the CR Tau compared to the CR group, the *Cdo* mRNA level was reduced, but not sufficiently to neutralize the difference in *Cdo* expression between Ad lib and CR groups. For *Bal*, gene expression was higher in the Ad lib Tau compared to the Ad lib group, and again, taurine addition diminished the difference between Ad lib and CR (Figure S1G).

Similar to the liver, supplementing taurine raised the levels of free taurine (Figure 2L) and multiple taurine conjugates (Figure 2M) in the plasma of Ad lib Tau and CR Tau mice compared to their respective control groups (statistical significance marked in white) (Figure 2K–L).

In the feces, both the Ad lib Tau and CR Tau groups showed increased concentrations of taurine (Figure 2N) and its conjugates (statistical significance indicated in black) (Figure 2O) compared to respective controls. However, the levels in Ad lib Tau groups were strikingly higher compared to any of the groups, implying that the previously described enhanced efficiency of taurine absorption in CR animals [9] is also maintained during taurine surplus, whereas Ad lib animals eliminate excess taurine with feces.

## 4 Discussion

In summary, the current study sought to challenge the CR characteristics associated with increased intestinal levels of taurine and its conjugates by administering either a diet that restricted taurine or providing taurine supplementation. The restriction or supplementation of taurine resulted in greater changes in hepatic and plasma compared to the intestinal taurine levels. The expression of GSTs in the intestine seemed to be more influenced by CR, while GST activity was more closely aligned with taurine‐GSH levels. However, in the liver, taurine‐GSH levels mirrored changes in taurine concentration in mice that received taurine supplementation, whereas GST activity remained unchanged. BA levels and composition were significantly impacted by CR but only moderately by dietary taurine. CR strongly influenced taurine uptake within the GI tract, particularly evident in mice given taurine supplementation.

In our study, LTD had a significantly greater effect on taurine and its conjugates in the liver than in the small intestine. A previous report using an LTD with a similar composition to the one used here found that rats exhibited decreased levels of taurine not only in the liver but also in the small intestine, blood, kidney, muscle, brain, and spleen [38]. Interestingly, in our experiments, similar to standard CR conditions, CR LTD mice exhibited increased levels of taurine and its conjugates in the ileum and plasma compared with Ad lib LTD controls. Moreover, while CR mice plasma displayed a reduction in taurine conjugate levels upon LTD, the impact of CR LTD was primairly on conjugate composition rather than on overall levels. Conversely, CR LTD did not show a further reduction in fecal taurine levels, which are typically low in CR conditions [9].

Taurine supplementation affected taurine levels in the ileum mucosa but had a much more pronounced impact on the liver and plasma taurine concentration. In a previous report, the implementation of a high‐taurine diet resulted in increased taurine levels in the plasma, liver, small intestine, kidney, muscle, and lung [38]. On the contrary, Sturman et al. reported that supplementing taurine has little effect on the concentration of taurine in the various tissues, with the exception of the liver and plasma [39]. In fact, in Sturman's study, liver taurine content was significantly influenced by dietary supplementation; thus, although the liver is a primary organ synthesizing taurine, it was hypothesized that the taurine levels in the liver are most influenced by changes in extracellular conditions, including diet [39]. Importantly, in mice and rats, the biosynthesis of taurine is regulated by the availability of taurine in the diet [40, 41]. Notably, humans demonstrate a much lower capacity to synthesize taurine compared to mice and rats [41]; thus, the presented results cannot be directly extrapolated to other species.

As mentioned, both the restriction and supplementation of taurine had a greater effect on taurine and taurine conjugate levels in the liver than in the small intestine. This may be linked to the fact that the intestine serves to take up and transport nutrients rather than accumulate them. Furthermore, modulation of dietary taurine levels did not alter the CR response in the intestine, for example, in terms of gene expression regulation. Notably, dietary taurine appears to elicit different outcomes compared to the CR‐related increase in taurine levels, as CR elevates taurine concentration in the intestinal mucosa but not in the liver, while dietary taurine primarily targets the liver and not the intestine. Therefore, distinct mechanisms regulate dietary versus restriction‐related taurine homeostasis

Recently, we have shown that GSH can spontaneously create conjugates with taurine and the rate of the reaction depends on both GSH and taurine concentration [13]. That is likely why, in the liver, where the concentrations of GSH as well as taurine are relatively high, their conjugation is more likely to occur non‐enzymatically. This, in turn, could explain why we previously discovered an increased mRNA expression and activity of GSTs in the CR intestine but not in the liver [9]. Notably, supplementation of taurine does not influence hepatic GST activity, further confirming a non‐enzymatic conjugation in the liver. Whereas in the small intestine, even upon taurine surplus, the reaction still relies on the activity of GSTs, likely reflecting a relatively low concentration of GSH. Notably, the patterns observed in the intestinal mucosa suggest that CR‐related taurine or other CR‐mediated factors, but not dietary taurine, impact GST gene expression. While the intestinal enzymatic activity of GSTs, as well as levels of taurine‐GSH, correspond more closely to changes in taurine levels and not GSTs gene expression.

Previously, Satsu H et al. [38] showed that intestinal Slc6a6 expression and taurine uptake are not affected by either a high‐taurine diet or LTD. Additionally, taurine transporters are saturable [42], which is likely why we observed that supplemented taurine is largely excreted with feces in ad libitum‐fed animals. Notably, the expression and activity of renal epithelial Slc6a6 is regulated by dietary sulfur amino acids, for example, LTD upregulates and high‐taurine diet down‐regulates it in kidneys [38, 43, 44, 45]. Consequently, in animals fed LTD, urinary taurine and fractional excretion of taurine is reduced compared to control, while taurine supplementation increases it [44]. Importantly, as we reported, during CR, the expression of intestinal Slc6a6 and taurine uptake increases [9], resulting in a reduced amount of taurine in the colon and feces. Now we show that CR has a stronger impact on taurine uptake than dietary taurine, and thus, the increase in taurine uptake also persists in CR animals upon taurine supplementation. This is well pictured by the fact that the difference in *Slc6a6* expression between Ad lib and CR intestine is also sustained upon taurine supplementation as well as by the striking difference in fecal taurine levels between ad libitum Tau and CR Tau groups. Importantly, the surplus of the consumed taurine was reported to be recovered almost quantitatively in both urine and feces [39], and in our most recent study [13], we measured that in CR animals the concentration of taurine in the kidneys and its secretion with urine is increased compared to ad libitum mice. Thus, the reduced fecal secretion of taurine in CR Tau compared to Ad lib Tau mice likely stems from redirection between urine versus fecal secretion.

The reason for enhanced hepatic synthesis of taurine during CR remains unveiled. One intriguing possibility is that since taurine stimulates the production of BAs [46, 47, 48, 49], this may be its main function in the CR liver, and its release in the intestine is only a derivative of this process. Taurine increases the mRNA expression and activity of CYP7A1 in mice [50, 51, 52] and in vitro HepG2 cells [48]. Similarly, in our study, we observe a modest increase in Cyp7a1 expression upon taurine supplementation accompanied by a increase in TCA and TUDCA concentrations. Importantly, there is no impact on *Shp*. Notably, in previous studies, taurine impacted CYP7A1 in mice fed a high‐fat/cholesterol diet; however, not in control chow‐fed mice [50, 51, 52]. Markedly, in the case of our study, the impact of taurine on BAs is rather mild, even though the amount of taurine used in our experiment is five to fifteen times higher compared to the studies investigating taurine in the context of BA [50, 51, 52]. Therefore, likely, the increase in taurine levels during CR is also not sufficient to stimulate BAs synthesis and other factors contribute to this regulation.

The translational relevance of these findings to human physiology must be considered carefully, given the substantial differences in taurine biosynthesis capacity between rodents and humans [39, 40, 41]. Humans have significantly lower taurine biosynthetic capacity compared to mice and rats, making dietary taurine intake more critical for maintaining tissue levels. Additionally, the BA conjugation profile differs substantially between species, with mice showing 95% taurine conjugation versus 30% in humans [7]. These differences suggest that dietary taurine interventions may have more pronounced effects in humans than observed in our mouse model, particularly regarding hepatic and plasma taurine levels. Future studies should incorporate human tissue models or clinical investigations to validate the therapeutic potential of taurine supplementation as a CR mimetic strategy.

Additionally, examining sex‐specific differences in taurine metabolism and CR responses represents a critical gap. The presented study utilized only male mice to maintain consistency with our previous publications on CR and taurine [9, 13, 14, 16, 36]. Sexual dimorphism in both taurine metabolism and CR responses has been documented in the literature, with females showing different taurine biosynthesis rates [53, 54] and distinct metabolic responses to CR [55, 56]. The use of male mice limits the generalizability of our findings, and future studies should examine whether the mechanistic independence between dietary and CR‐induced taurine regulation observed here also applies to female mice. A key consideration of this study is the 14‐day duration of the CR intervention. This timeframe was chosen based on our experience, which shows that CR‐induced taurine‐related changes are fully established by week 2 and remain stable thereafter. This protocol has been consistently used across our CR‐taurine publications [9, 13, 14, 16, 36], enabling direct mechanistic comparisons. While the protocol induces metabolic adaptations and effectively captures the taurine‐related CR outcomes, long‐term CR may reveal additional adaptive mechanisms involving tissue architecture remodeling, immune modulation, and cellular senescence [57, 58]. Whether the mechanistic independence between dietary and CR‐induced taurine regulation persists over prolonged restriction remains to be determined.

While some CR protocols incorporate micronutrient‐matched diets to isolate caloric effects from potential micronutrient deficiency [59], we employed a non‐supplemented approach. The main reason for this design is the formulation of the standard rodent chow, which provide nutrients in excess of the minimal requirements, ensuring adequate micronutrient intake even with 20% CR [60]. Furthermore, our CR protocol involved a modest 20% restriction applied over a short 14‐day period, generally considered mild and insufficient to induce clinically significant side effects [61]. Finally, this protocol reflects dietary restriction in humans, where CR is rarely accompanied by compensatory micronutrient supplementation. However, we acknowledge this as a limitation, as it cannot be ruled out that subtle micronutrient fluctuations contributed to the observed changes in taurine metabolism. Future research using diet plans matched for micronutrients, along with plasma micronutrient profiling, could provide more definitive evidence and strengthen causal links between dietary taurine adjustments and their effects, independent of overall nutritional variation.

Another limitation of our study is the difference in housing conditions between the experimental groups. Unlike the ad libitum groups, CR animals were housed individually, thereby preventing aggression and ensuring equal food distribution. This design may introduce confounding variables related to social isolation stress. Although it is unlikely to influence taurine metabolism, future studies employing parallel housing conditions across all experimental groups should assess the isolated contribution of housing to CR‐induced metabolic changes.

Other key remaining questions requiring investigation include the dose‐response relationship between taurine supplementation and CR‐mimetic effects across different metabolic outcomes, the temporal dynamics of taurine homeostasis changes during long‐term CR protocols, and the functional significance of tissue‐specific taurine‐glutathione conjugates in cellular protection mechanisms. In summary, modulating taurine levels through dietary supplementation or restriction did not affect the CR‐related increase in the intestinal taurine levels and GST expression. Moreover, CR increases taurine concentration in the intestine but not in the liver, whereas dietary taurine primarily targets the liver rather than the intestine. Thus, dietary taurine appears to have a distinct impact from the CR‐related increase in taurine levels and in both cases, the outcomes are tissue‐dependent. Overall, our study contributes to a nuanced understanding of the intricate interplay between dietary factors, metabolic regulation, and tissue‐specific responses in taurine metabolism. This study highlights the importance of tailoring dietary interventions to target specific tissues, potentially leading to more effective strategies for managing metabolic health and obesity [62].

## Conflicts of Interest

The authors have no conflict of interest to declare.

## Declaration of Generative AI and AI‐Assisted Technologies in the Writing Process

No AI and AI‐assisted technologies were used in the writing process.

## Supporting information




**Supporting File**: mnfr70414‐sup‐0001‐SuppMat.pdf.

## Data Availability

Raw data generated for this study are available from the corresponding author on request.
