# Supplementary material for: Caloric Restriction and Dietary Taurine Regulate Taurine Homeostasis Through Distinct Tissue‐Specific Mechanisms in Mice
Source: Mol Nutr Food Res. 2026 Feb 12;70(3):e70414. doi: 10.1002/mnfr.70414 (PMC12896083; doi:10.1002/mnfr.70414)
Supplement: Supplementary file 1 — Supporting File: mnfr70414‐sup‐0001‐SuppMat.pdf. [file MNFR-70-e70414-s001.pdf]

Supplementary data

Supplementary table 1: Composition of diets used in experiment 1.

g/100g	LTD	Ctrl
Casein	10	10
Corn starch	52.3	51.8
Sucrose	10	10
Maltodextrin	12.5	12.5
Soybean oil	5	5
Fibre mix	3	3
Vitamin mix	1	1
Mineral mix	6	6
Choline	0.2	0.2
Taurine	0	0.5

Supplementary table 2: Composition of diets used in experiment 2

	V153x<br>R/M-H auto
Crude protein (%)	19
Crude fat (%)	3.3
Crude fibre (%)	4.9
ME (MJ/kg)	12.9
CP/ME (g/MG)	14.7

Supplementary table 3: qRT-PCR primers

Gene ID	Forward	Reverse
Bal	TGTGTGTGAAGGAACCTGGA	ACCCGGACAACTTTGTGAAG
Cdo	GGGGACGAAGTCAACGTGG	ACCCCAGCACAGAATCATCAG
Cyp7a1	TGTCTGCGAGGGCTGGAGCA	CCAGCCTGGGATGCTATGGGC
Eef1a1	CCTGGCAAGCCCATGTGT	TCATGTCACGAACAGCAAAGC
Mgst1	CCTTCTCCCTGGATTCAGTCAT	TCGGCCATGCTTCCAATCTT
Shp	GAAAGAAAGATGGCCGGGGA	CCGCCAAGCGAAATAACTTTG
TauT	GCACACGGCCTGAAGATGA	ATTTTTGTAGCAGAGGTACGGG

Supplementary figure 1

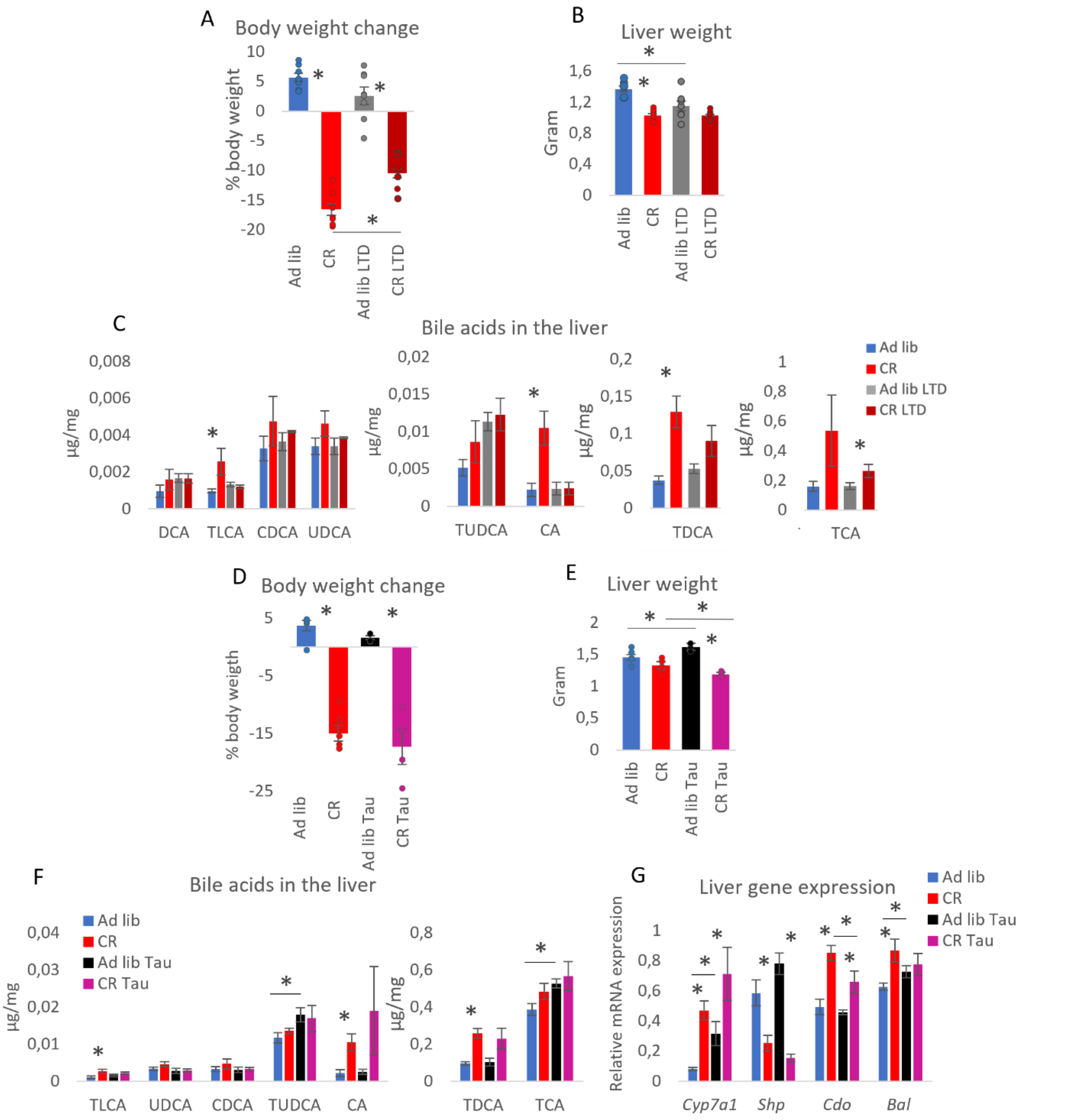

Body (A) and liver weight (B), as well as the concentration of hepatic bile acids (C), were measured for control and LTD-fed ad lib and CR mice. Correspondingly, body weight (D) and liver weight (E), the concentration of hepatic bile acids (F) as well as expression of genes connected with bile acids (*Cyp7a1* and *Shp*) and taurine (*Cdo*) synthesis, and conjugation of taurine with bile acids (*Bal*), were measured in the liver of control and taurine-supplemented (Tau) animals (G). Bile acids: CA: cholic acid; CDCA: chenodeoxycholic acid; DCA: deoxycholic

acid; TCA: taurocholic acid; TDCA: taurodeoxycholic acid; TLCA: taurolithocholic acid; TUDCA: tauroursodeoxycholic acid; UDCA: ursodeoxycholic acid. ANOVA was applied to assess statistical differences between the groups; * represents statistical significance. Error bars represent ±SEM.
